# Detection and Surgical Approach to Pheochromocytoma: A Case Report

**DOI:** 10.7759/cureus.55504

**Published:** 2024-03-04

**Authors:** Alexis Jared Paz-López, Carlos Ignacio Rafael-Pérez, Brenda Aurora Llanos-Salas, Paola Saskia Castañeda-Anaya, Samuel Rodrigo Gómez-Arenas, Yamir Ahmed Nacud-Bezies

**Affiliations:** 1 Departamento de cirugía digestiva y endocrina, Unidad Médica de Alta Especialidad No. 25, Monterrey, MEX

**Keywords:** neuroendocrine neoplasm, hypertension and pheochromocytoma, endocrine neoplasms, adrenal surgery, adrenal pheochromocytoma

## Abstract

Pheochromocytomas are neuroendocrine tumors that produce, store, and secrete catecholamines. They are found in the chromaffin tissue of the adrenal medulla and manifest clinical symptoms by producing an excessive amount of one or more catecholamines, such as dopamine, adrenaline, and noradrenaline, as well as their inactive metabolites, such as metanephrine, normetanephrine, and 3-methoxytyramine. This paper is the case report of a 53-year-old male patient with diabetes and hypertension who has been experiencing symptoms such as night sweats, frequent colds, weight loss, reduced appetite, and generalized anxiety. The patient presented with pelvic pain and sought medical attention, leading to an abdominal MRI scan that revealed a right adrenal mass. The patient’s plasma metanephrine levels were found to be four-fold higher than the normal range. A contrast CT scan of the abdomen and pelvis revealed a right adrenal gland with increased dimensions and well-defined edges. A diagnosis of right adrenal pheochromocytoma was made. The patient underwent a right laparoscopic adrenalectomy, which resulted in a reduction in metanephrine levels and normal blood pressure readings. The patient presented a favorable clinical evolution in the post-surgical period, for which it was decided to be discharged home.

## Introduction

Pheochromocytomas are infrequent neuroendocrine tumors that originate from the chromaffin tissue of the adrenal medulla, which is derived from neural crest cells. These tumors possess the ability to produce, store, and secrete catecholamines. Catecholamines undergo partial or complete conversion into inactive metabolites through the enzymatic activity of catechol-O-methyltransferase found within tumor cells. The presence of a greater than four-fold elevation in catecholamine metabolites is highly indicative of a potential diagnosis of pheochromocytoma, a condition that warrants further investigation using imaging techniques [1,2].

These tumors manifest clinical symptoms by producing an excessive amount of one or more catecholamines, namely dopamine, adrenaline, and noradrenaline, as well as their inactive metabolites, such as metanephrine, normetanephrine, and 3-methoxytyramine [1]. The majority, around 80-85%, of pheochromocytomas are situated within the adrenal medulla, whereas the remaining 15-20% are found in extra-adrenal locations [3]. There is a wide spectrum of possible presenting symptoms, one of which includes the classic triad of headaches, palpitations, and profuse sweating [4]. However, hypertension is the most common finding, occurring in 80-90% of the triad of symptoms. The triad is seen in only 25% of patients with pheochromocytomas. Other symptoms may include paleness, nausea, vomiting, constipation, hot flashes, weight loss, weakness, fever, orthostatic hypotension, chest and/or abdominal pain, hyperglycemia, and anxiety [1].

The diagnosis of pheochromocytomas necessitates the administration of a catecholamine excessive release test and the documenting of the tumor’s anatomical characteristics. The mean sensitivity of an elevation in plasma fractionated metanephrines is 97%, while its specificity is 93%. Functional adrenal incidentalomas are frequently observed, although they still constitute a minority (perhaps less than 10%) of all cases. The primary difficulty lies in accurately diagnosing these lesions. In the context of a suspected adrenal gland tumor, it is advisable to conduct tests to exclude the presence of pheochromocytoma [5]. There is the rule of 10, which refers to the following: 10% are extra-adrenal, 10% occur in children, 10% are multiple or bilateral, 10% recurrence after surgery, 10% are malignant; 10% are familial, and 10% are discovered as adrenal incidentalomas [3]. It must be considered that up to 25% of all pheochromocytomas are discovered incidentally, and according to the series, up to 8% of adrenal incidentalomas are pheochromocytomas [6].

Malignancy is observed in a minimum of 10% of sympathetic pheochromocytomas, with variations in malignancy rates depending on the individual’s familial background. According to available data, the prevailing locations of metastasis in individuals diagnosed with pheochromocytomas include local and distant lymph nodes (80%), skeletal system (72%), liver (50%), and lungs (50%) [7].

In this case report, we are pleased to present a patient with the confirmed diagnosis of pheochromocytoma, characterized by unusually elevated levels of metanephrines. This unique case highlights the presence of the clinical condition in question and underlines the notable magnitude of metanephrine concentrations, generating a clinical scenario of particular interest and relevance.

## Case presentation

Patient information

This paper is a case report of a 53-year-old male patient who has been diagnosed with diabetes mellitus and hypertension for a duration of 15 years. The patient’s adherence to therapy has been inconsistent. The patient reported night sweats, frequent colds, significant weight loss of 8 kg, reduced appetite, and generalized anxiety, which commenced approximately one year ago. Following this, the patient presented with pelvic pain and sought medical attention from a specific healthcare professional who ordered a thoracic-abdominal magnetic resonance imaging (MRI) scan. The abdominal MRI scan indicated the existence of a right adrenal mass measuring 5 × 4.5 cm. This mass exhibited higher intensity than the liver in T2 imaging and was of similar intensity in T1 imaging. Additionally, the presence of localized central hypointensities, which appeared hyperintense in T2 imaging, was observed.

The patient was then referred to the endocrinology department for assessment, during which the patient’s plasma metanephrine levels were measured and found to be four-fold higher than the established normal range. Due to this rationale, he was assigned to our division for the purpose of assessment.

Clinical findings

During the physical examination, it was observed that the abdomen exhibited a rounded shape due to the presence of adipose tissue. The abdomen felt soft and could be depressed upon palpation. No palpable tumors were detected, and there were no indications of peritoneal irritation. Peristalsis was present. Also, both hind extremities were found to be symmetrical.

Diagnostic assessment

**Figure 1:**
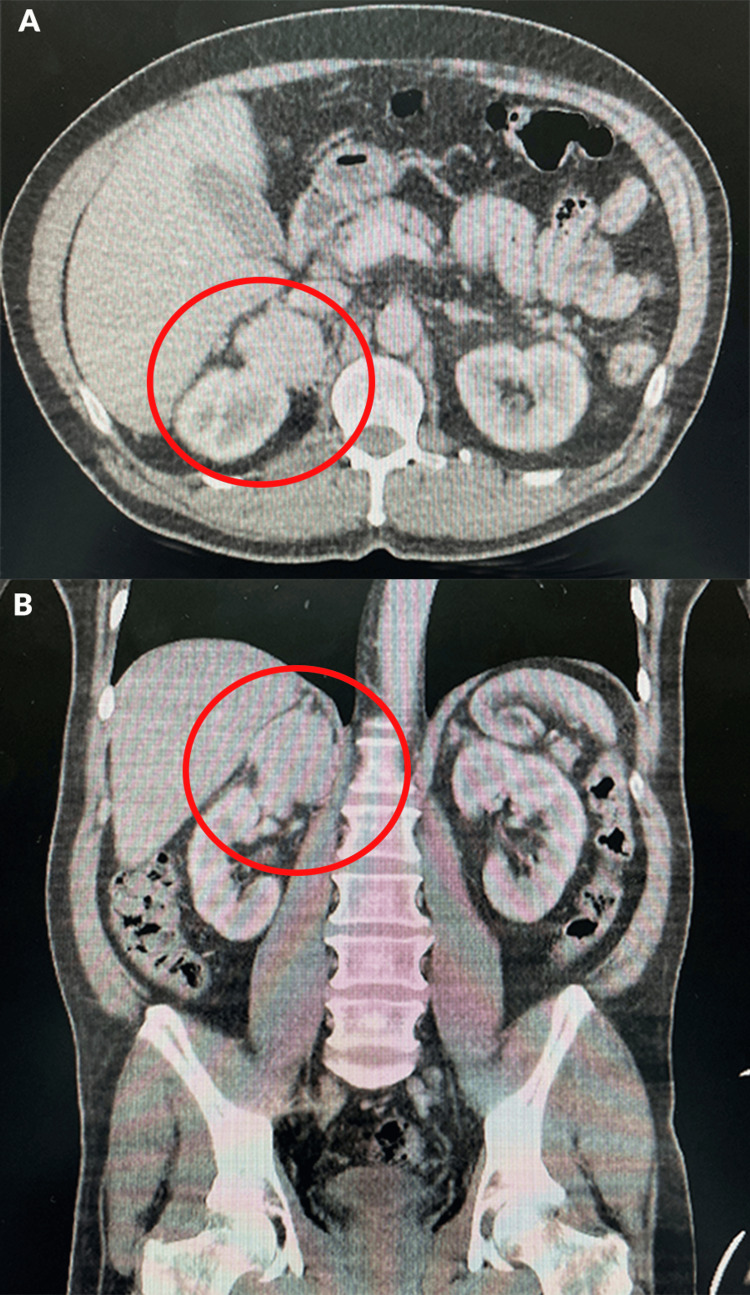
Contrast tomography (washout) A right adrenal tumor is observed with characteristics suggestive of adenoma due to the washing phase, and the left adrenal gland is observed within normal limits. (A) Axial section (B) Coronal section

The study protocol was started, and laboratory studies were requested. Also, a contrast CT scan of the abdomen and pelvis (washout) was requested, reporting a right adrenal gland with increased dimensions at the expense of a mass with well-defined edges, which measured 5. 1 × 4.7 × 6 cm, with a density of 32 UH in the simple phase, 103 UH in the late arterial post-contrast phase, and 45 UH in the washout phase, with an absolute washout of 81.7% and a relative washout of 56.3% (Figure 1). A diagnosis of right adrenal pheochromocytoma was made. The patient’s laboratory results are shown in Table 1.

**Table 1:** Patient’s laboratory results

Lab test	Patient result	Normal Range
Cortisol	2.60 mcg/dL	6-8 am, 10-20 mcg/dL
HbA1c	6.5%	4-5.6%
Glucose	308 mg/dL	70-100 mg/dL
Total cholesterol	277 mg/dL	<200 mg/dL
Creatinine	1.5 mg/dL	0.7-1.3 mg/dL
Aldosterone	39.10 ng/dL	3.1-35.4 ng/dL
Renine	149 5.38 ng/mL/h	0.167-5.38 ng/mL/h
Total plasmatic metanephrines	9218 pg/mL	0-62 pg/mL

Therapeutic interventions

**Figure 2:**
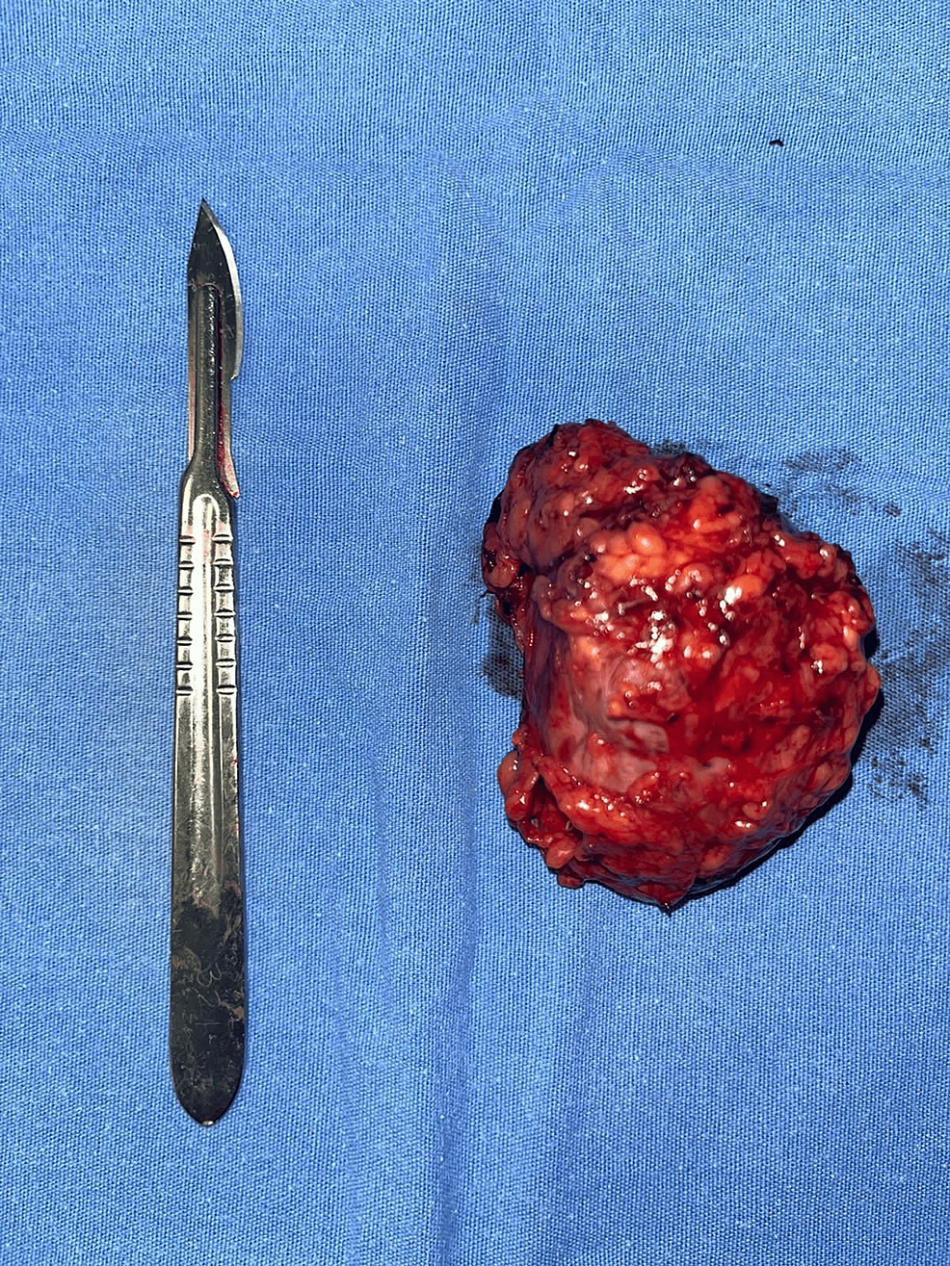
Tumor from the abdominal cavity dependent on the right adrenal gland

**Figure 3:**
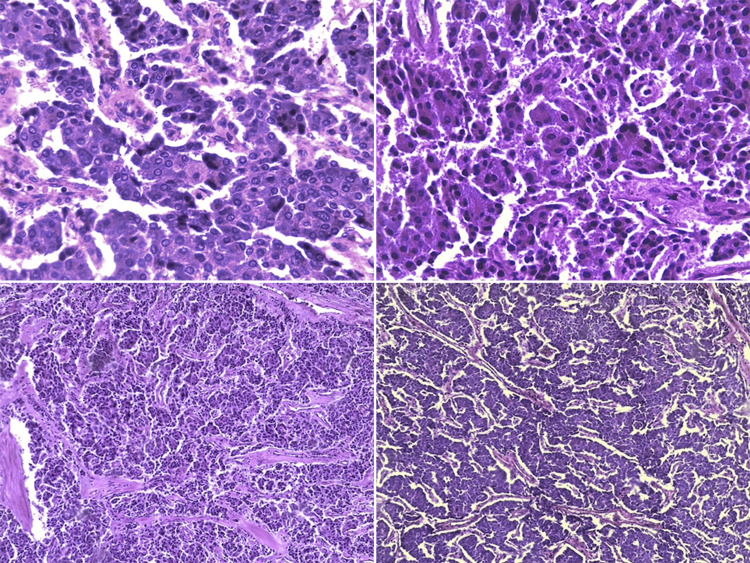
Histological section showing adrenal gland with enterochromaffin cells

Preoperatively, the patient received a course of antihypertensive therapy consisting of phenoxybenzamine and losartan for a duration of 10 days. This treatment successfully resulted in blood pressure readings that fell within the normal range, demonstrating satisfactory control. The patient underwent right laparoscopic adrenalectomy with a transabdominal approach, extracting the right adrenal gland measuring 8 × 7 × 5 cm, without the presence of abnormal adhesions, and a single right adrenal vein measuring 1.5 cm toward the posterolateral aspect of the inferior vena cava (Figure 2). A total of 400 mL of bleeding and a duration of the procedure of 165 minutes were reported. The sample was then sent to pathology, which reported an adrenal gland with enterochromaffin cells (Figure 3).

Follow-up and outcomes

The patient presented a favorable clinical evolution in the post-surgical period, for which it was decided to be discharged home. The patient attends a consultation for endocrine and digestive surgery one month following the surgical procedure. The patient presents laboratory studies indicating a reduction in metanephrines levels (210 pg/mL). Additionally, the patient’s blood pressure readings were within the normal range, and he did not exhibit any symptoms. The surgical wounds exhibited satisfactory healing, prompting the continuation of annual appointments for clinical and biochemical examination throughout the initial five-year period, followed by biannual appointments until the whole 10-year monitoring period was completed.

## Discussion

The diagnosis of pheochromocytoma necessitates the administration of an excessive catecholamine release test and the anatomical description of the tumor. The average sensitivity of an elevation in plasma metanephrines (metanephrine and normetanephrine) is 97%, while its specificity is 93% [4]. Functional adrenal incidentalomas are frequently observed, although they likely constitute less than 10% of the overall cases. However, the primary difficulty lies in accurately diagnosing these lesions. If there is a suspicion of a tumor in the adrenal gland, it is advisable to conduct testing to exclude the possibility of pheochromocytoma [5,8]. In this particular case, the identification of total serum metanephrine levels over 9000 units served as a diagnostic marker for the presence of a pheochromocytoma. Consequently, the investigation proceeded with an imaging study.

While existing studies have established a prevailing trend of elevated metanephrine levels in male patients aged 50-65 years [9,10], our patient’s case stands out as a unique and intriguing anomaly in the literature. Remarkably, no other report has documented metanephrine levels reaching the highs observed in our patient’s case. This observation raises compelling questions about the factors contributing to such a distinctive profile. Moreover, it prompts a critical examination of the potential correlation between these unusually elevated metanephrine levels and the specific clinical manifestations exhibited by the patient. This nuanced perspective encourages further exploration into the underlying mechanisms that might drive such atypical presentations, potentially opening new avenues for research and contributing to a deeper understanding of the complex interplay between metanephrine levels and clinical outcomes in diverse patient populations.

Alpha-adrenergic antagonists, such as phenoxybenzamine, are advised for usage at the preoperative level [11]. The administration of this medicine can be initiated within a time frame of seven to 14 days before the surgical procedure, resulting in a notable enhancement in the stabilization of blood pressure measurements. This intervention offers the advantage of decreased pre-load and cardiac stress. Furthermore, the utilization of selective beta-blockers results in the manifestation of negative inotropic and chronotropic characteristics. Additionally, these blockers prevent excessive oxygen consumption at the cellular level of the heart, hence enhancing cardiac performance [11]. The patient presented was receiving antihypertensive therapy with phenoxybenzamine and losartan, resulting in satisfactory preoperative management aimed at mitigating the patient’s susceptibility to cardiovascular complications.

The guidelines suggest that surgical intervention for individuals diagnosed with pheochromocytoma should be restricted to specialized medical facilities and conducted by surgeons who possess expertise in adrenal surgery, both open and laparoscopic procedures. Additionally, it is advised that these physicians have a minimum annual caseload of 15 adrenalectomies, encompassing both benign and malignant cases. The surgical excision of pheochromocytoma is considered the fundamental approach to its treatment. Currently, laparoscopic adrenalectomy is widely regarded as the preferred surgical approach for the majority of small- to medium-sized adrenal abnormalities. Numerous studies have provided evidence supporting the benefits of shorter hospital stays, decreased pain levels, reduced morbidity rates, and enhanced recovery outcomes [5,12].

The utilization of laparoscopic adrenalectomy may be deemed appropriate for adrenal tumors that measure 6 cm or smaller, exhibit radiological indications of malignancy, and do not present any indications of local invasion. The recommendation for open resection is based on the higher risk associated with bigger tumors. It is advisable to make the option to transition to an open treatment in a timely manner, prior to any potential rupture of the tumor capsule. Surgical excision is advised in cases where there is a growth of at least 20% in size, accompanied by an increase in diameter of no less than 5 mm during the specified time frame [5,12,13]. The posterior laparoscopic approach offers a means of directly accessing the adrenal gland while circumventing the intra-abdominal canal. Moreover, it enables the performance of bilateral treatments without necessitating patient movement. One of the drawbacks associated with the posterior retroperitoneoscopic method is the limited working space it provides. This can provide challenges when attempting to extract larger tumors and may be particularly tough in individuals with a high body mass index (BMI). In addition, it has been demonstrated that the transperitoneal method is both safe and efficacious. Robotic surgery presents a broader spectrum of motion, enhanced precision, and stereoscopic visual capabilities in comparison to conventional laparoscopic surgery [5,14].

It is advisable to do postoperative monitoring by the utilization of laboratory and imaging examinations. In the context of urine or plasma metanephrine levels, it is advisable to do a subsequent assessment within a time frame of two to six weeks following surgical recuperation in those who exhibited heightened levels before the surgical intervention. Furthermore, it is recommended that an imaging examination be conducted three months post-surgery to assess the presumed completeness of the procedure. In conclusion, it is advisable to assess the levels of metanephrine in plasma or urine and conduct an imaging examination on a yearly basis [7]. The occurrence rate of novel events is quite low, approximately one per 100 person-years. However, it is noteworthy that over 40% of these new events are characterized by malignant recurrences. Additionally, it is important to acknowledge that new events might manifest even after a period of five years without any prior occurrences [7].

## Conclusions

Pheochromocytomas are a medical disorder characterized by a relatively low prevalence. The detection of a tumor in the adrenal gland by imaging methods requires us to carry out biochemical tests to determine its origin. The patient’s clinic presentation suggests a potential adrenal gland condition, necessitating a comprehensive evaluation involving differential diagnoses through laboratory or imaging studies. Additionally, it is crucial to inquire about the patient’s cancer history, as the tumor could potentially be a result of metastasis from another source, such as the kidney. The diagnosis was achieved based on the patient’s history of recurring hypertensive crises that necessitated hospitalization. An MRI study revealed the presence of a tumor mass, and subsequently, a plasma metanephrine level was found to be more than four times higher than the normal value. These findings led to the presumptive diagnosis of pheochromocytoma, and surgical resection was scheduled. The method, as substantiated by scholarly sources, is widely regarded as the principal therapeutic intervention for individuals diagnosed with functional tumors who manifest symptomatic manifestations.
